# Non-bacterial Thrombotic Endocarditis Related to Squamous Cell Carcinoma of the Cervix

**DOI:** 10.7759/cureus.42128

**Published:** 2023-07-19

**Authors:** Megan Gromke, Bryce Beard

**Affiliations:** 1 Oncology, Edward Via College of Osteopathic Medicine, Blacksburg, USA; 2 Radiation Oncology, Rapides Regional Medical Center, Alexandria, USA

**Keywords:** thrombosis, squamous cell, cervical cancer, marantic endocarditis, non-bacterial thrombotic endocarditis

## Abstract

We report the case of a 51-year-old woman who presented with multiple thrombotic events, including deep vein thrombosis, extensive pulmonary embolisms, myocardial infarction, and multiple ischemic strokes suggesting cardiogenic embolization. Recent history was significant for locally advanced squamous cell carcinoma of the cervix. Echocardiogram revealed large aortic valve vegetations in the absence of evidence of infectious endocarditis consistent with the diagnosis of non-bacterial thrombotic endocarditis (NBTE). This case is a rare presentation of NBTE associated with squamous cell carcinoma of the cervix.

## Introduction

Hypercoagulability is a frequent paraneoplastic complication of malignancy, and it affects approximately 15% of cancer patients during their illness [1]. The most common thrombotic events are venous thromboembolism and migratory superficial thrombophlebitis. Less common presentations are disseminated intravascular coagulation, thrombotic microangiopathy, and arterial thrombosis. Rarely do patients present with non-bacterial thrombotic endocarditis (NBTE) [1]. NBTE, formally known as marantic endocarditis, is associated with inflammatory states such as autoimmune diseases and malignancy [2,3]. Lung, pancreatic, gastric cancer and adenocarcinomas of an unknown primary site are most commonly associated with NBTE [4]. A review of the literature failed to reveal any other cases of squamous cell cervical cancer associated with NBTE. Here, we present a unique case of a patient with squamous cell carcinoma of the cervix presenting with multiple thrombotic events, including deep vein thrombosis (DVT), extensive pulmonary embolisms (PE), ischemic strokes, and myocardial infarction in the context of NBTE.

## Case presentation

**Figure 1:**
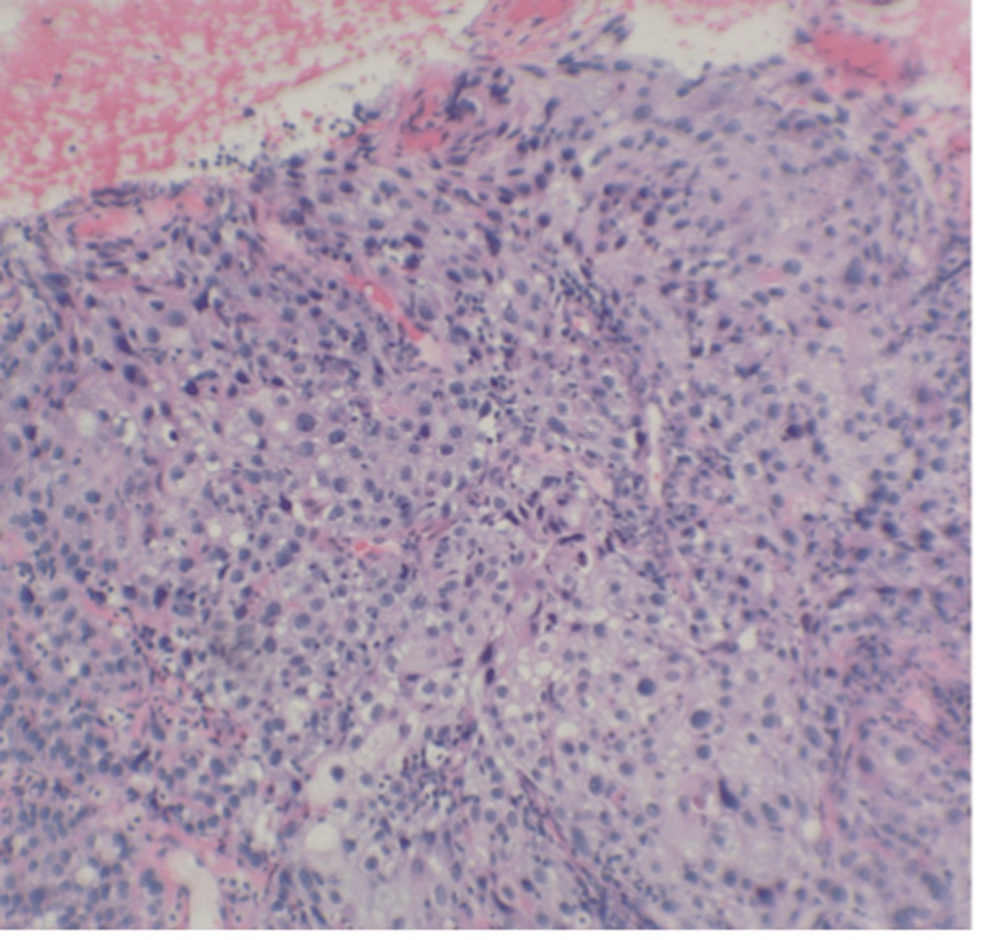
Biopsy specimen of the cervix. 40x view of a hematoxylin and eosin stain of the cervix showed poorly differentiated squamous cell carcinoma.

**Figure 2:**
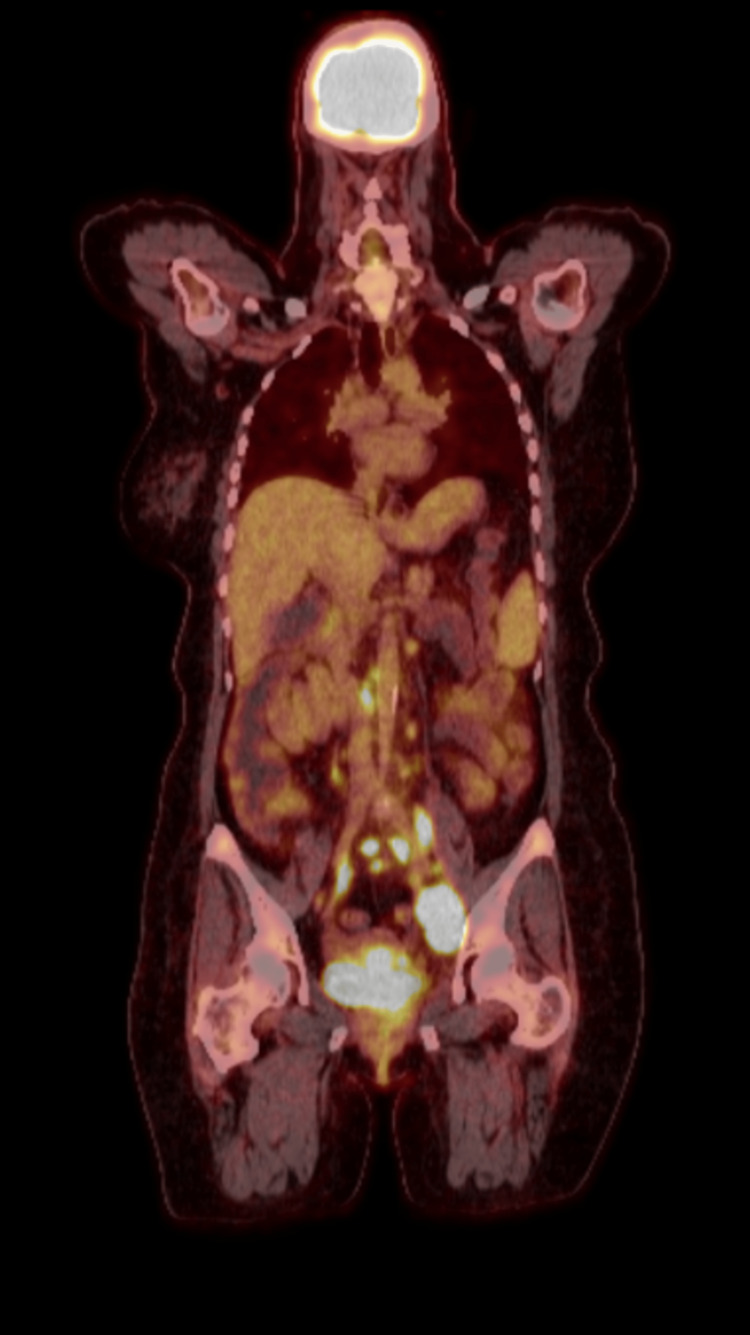
Coronal positron emission tomography (PET) scan and a computed tomography (CT) scan of the skull to thigh. Coronal view of fluorodeoxyglucose (FDG)-positron emission tomography (PET) scan of the patient showed FDG uptake in the cervix and multiple pelvic and para-aortic lymph nodes with extension to renal vasculature.

A 51-year-old woman, non-smoker with a history of diabetes, hypertension, and no reportable autoimmune disease, presented to the emergency room with unintentional weight loss and left lower extremity (LLE) pain. She had no previous medical or family history of venous thrombosis, recent immobility, or vascular injury. Laboratory evaluation showed white blood cell count 7.4/uL, hemoglobin 9.6 g/dl, hematocrit 32.9 L/L, mean corpuscular volume 79 μm^3^, platelet 328 × 10^9^/L, prothrombin time 12.7 seconds, international normalized ratio 1.1, partial thromboplastin time 27.1 seconds, c-reactive protein 19.7 mg/L, and carcinoembryonic antigen 11.6 ug/L. The lower extremity Doppler was positive for thrombosis, and the computed tomography pulmonary angiogram showed extensive bilateral pulmonary emboli. A computed tomography (CT) of the chest, abdomen, and pelvis revealed a 5 cm uterine cervical mass with multiple pathologically enlarged lymph nodes extending to the aortic bifurcation. On physical examination, she appeared generally well. She had left lower extremity edema. There were no murmurs on cardiac auscultation. A speculum exam revealed a friable mass in the uterine cervix involving the proximal vagina and extending half the distance to the vaginal introitus. On rectovaginal exam, there was evidence of bilateral parametrial invasion with extension to the left pelvic sidewall. The cervical mass was biopsied (Figure 1), and pathology showed poorly differentiated squamous cell carcinoma. Subsequent esophagogastroduodenoscopy (EGD) and colonoscopy were both negative for malignancy. Prior to discharge, a thrombectomy was performed on the LLE, and the patient was placed on rivaroxaban. Positron emission tomography (PET) CT (Figure 2) showed PET avid disease in the cervix as well as in multiple pelvic and para-aortic lymph nodes extending up to the renal vessels. There was no evidence of distant metastases. FIGO stage IIIC2 (AJCC 8^th^ edition T3bN2M0). 

**Figure 3:**
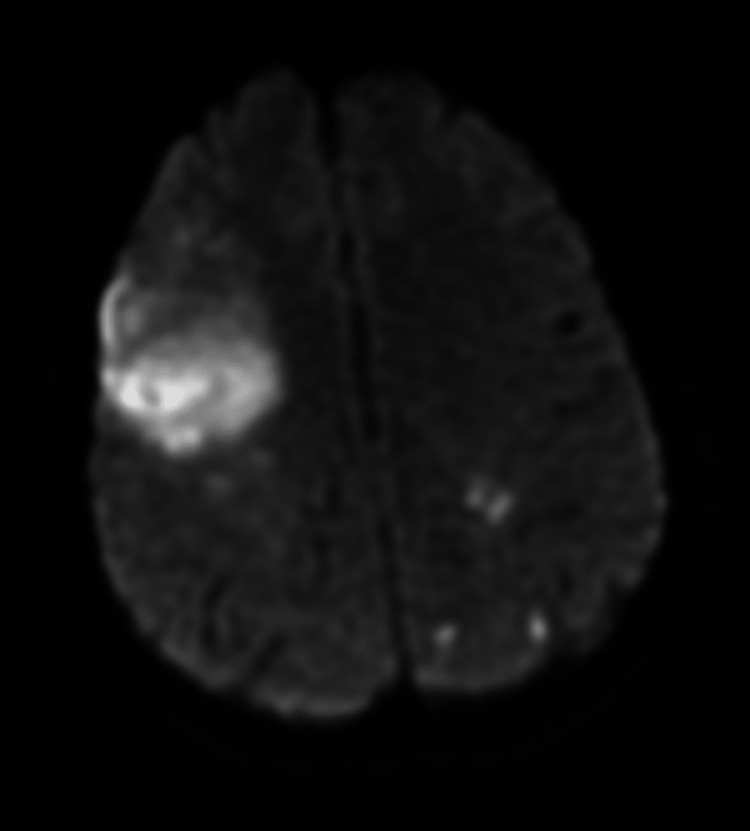
Axial diffusion-weighted image. Axial images show an acute right superior frontal and anterior superior temporal cerebrovascular accident with associated areas of diffuse small ischemic events.

**Figure 4:**
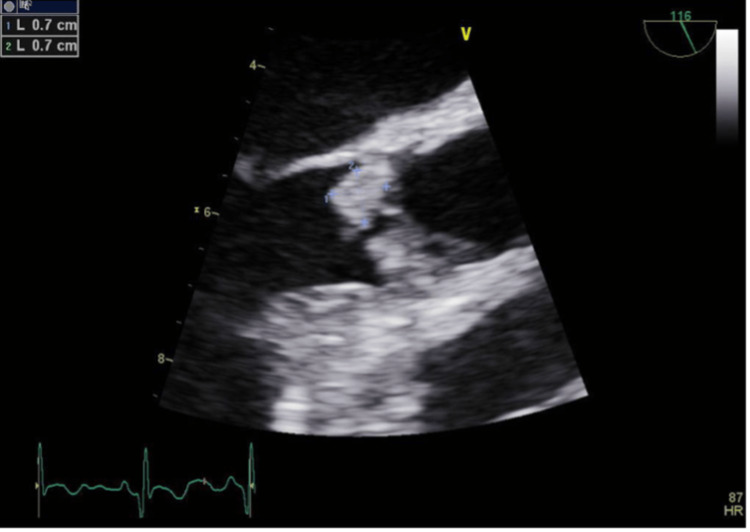
Transesophageal echocardiography (TEE). Transesophageal echocardiography showed large aortic valve vegetation with severe aortic regurgitation.

The patient was evaluated by radiation oncology and medical oncology, and concurrent chemoradiation therapy with weekly cisplatin was recommended. Before starting treatment, the patient presented to the emergency room with dysarthria, left-sided facial droop, and weakness. On physical exam, the patient was found to have profound left upper and lower extremity weakness and a soft diastolic murmur auscultated at the left upper parasternal border. CT head showed hypodensity in the left frontal lobe concerning vasogenic edema related to an acute infarct of the right frontal lobe without evidence of intracranial bleeding. Magnetic resonance imaging (MRI) brain (Figure 3) findings were consistent with an acute right superior frontal and anterior superior temporal cerebrovascular accident as well as areas of diffuse small ischemic events. Carotid Doppler was consistent with left-sided moderate disease (50-69% lumen narrowing). CT angiogram of the head and neck showed no high-grade stenosis or large vessel occlusion. Echocardiogram (ECHO) and transesophageal echocardiogram (TEE) (Figure 4) showed a large aortic valve vegetation with severe aortic regurgitation.

Throughout the hospitalization, the patient remained afebrile. Two sets of blood cultures were negative for infectious etiology. Empiric antibiotics were initiated without clinical improvement. Hospice was recommended secondary to her declining performance status and the profoundness of her thrombophilia. The patient died of her disease seven weeks after her initial presentation. The immediate cause of death is not certain, as our team did not see her after her discharge to hospice. However, we consider her thromboembolic disease to be a paraneoplastic manifestation of her cancer. Therefore, her death can be considered cancer-related. 

## Discussion

Malignancy-induced NBTE consists of sterile vegetations accumulating on cardiac valves with a preference for the mitral and aortic valves [4]. The pathogenesis of NBTE is not clearly understood [5]. The presumed mechanism involves the interaction between macrophages and malignant cells [1]. Cytokines released from malignant cells, such as interleukin-1 and tumor necrosis factor, activate the coagulation cascade resulting in damage to the endothelium causing platelet aggregation and thrombus formation [1,6]. The vegetations associated with NBTE tend to be friable because of the absence of cellular organization, causing them to have a high tendency for embolization [7].

The prevalence of NBTE is unknown because diagnosis usually occurs post-mortem [1]. NBTE should be suspected in cancer patients presenting with recurrent emboli and ischemic stroke [3]. No official diagnostic criteria exist for NBTE [8]. Diagnosis of NBTE is based on both clinical and echocardiographic findings with the exclusion of infectious etiology of the endocarditis [9]. Valvular destruction and new significant murmurs are uncommon in NBTE, distinguishing it from infectious endocarditis [6]. Vegetations associated with NBTE can be quite small and easily missed by transthoracic echocardiogram. Therefore, all patients require transesophageal echocardiogram to search for vegetations [9].

Cancer-related NBTE has a poor prognosis, typically associated with advanced malignancy [3]. Treatment of the underlying disease, as well as anticoagulation with intravenous heparin or low-molecular-weight heparin, is recommended by the American College of Chest Physician guidelines [10]. In NBTE, warfarin has been found to be ineffective in preventing recurrent thromboembolic events [6]. There is limited data to suggest the use of factor Xa and thrombin inhibitors in the setting of NBTE [8]. The use of valvular surgery is limited secondary to the overall poor functional status of most patients. 

## Conclusions

Our case illustrates a rare presentation of NBTE associated with locally advanced squamous cell carcinoma of the cervix in a middle-aged woman, non-smoker with no relevant family history or reportable autoimmune disease. Our patient presented with multiple thrombotic events, both clinical and echocardiogram findings consistent with NBTE, and infectious etiology was excluded as a cause of her embolic endocarditis. Our case suggests that NBTE associated with cervical cancer may be an under-reported paraneoplastic manifestation of cervical cancer and is associated with a poor prognosis. 
